# Supplementary material for: Social-behavioral insights in understanding tuberculosis transmission pattern during the COVID-19 pandemic period in Kuala Lumpur, Malaysia: The MyTBNet study protocol
Source: PLoS One. 2024 Sep 26;19(9):e0307921. doi: 10.1371/journal.pone.0307921 (PMC11426513; doi:10.1371/journal.pone.0307921)
Supplement: S1 Table — (PDF) [file pone.0307921.s001.pdf]

S1 Table. List of 16 government TB treatment centres by health administrative area in Kuala Lumpur

Health administrative area	Num.	Name of TB treatment centre
Lembah Pantai	1	Tanglin health clinic
	2	Petaling Bahagia health clinic
Cheras	3	Cheras health clinic
	4	Cheras baru health clinic
	5	Sungai Besi health clinic
	6	Bandar Tun Razak health clinic
	7	Sungai Besi health clinic
	8	Salak Selatan health clinic
Kepong	9	Jinjang health clinic
	10	Batu health clinic
	11	Sentul health clinic
	12	Segambut health clinic
Titiwangsa	13	Kuala Lumpur health clinic
	14	Setapak health clinic
	15	Kampung Pandan health clinic
	16	Dato’ Keramat health clinic
